# A Curious Case of Pediatric Granulomatosis and Polyangiitis

**DOI:** 10.7759/cureus.82968

**Published:** 2025-04-25

**Authors:** Diya E Viju, Daya Mani Jacob, Rehna K Rahman, Shaik Irfan Basha, Ghulam Mujtaba

**Affiliations:** 1 Pediatrics, Burjeel Medical City, Abu Dhabi, ARE; 2 Nephrology, Burjeel Medical City, Abu Dhabi, ARE; 3 ENT, Burjeel Medical City, Abu Dhabi, ARE; 4 Paediatric Pulmonology, Burjeel Medical City, Abu Dhabi, ARE

**Keywords:** fever, granulomatosis with polyangiitis (gpa), noncaseating granuloma, orbital proptosis, pediatrics & child health, wegner’s granulomatosis

## Abstract

Granulomatosis with polyangiitis (GPA), formerly known as Wegener’s granulomatosis, is a rare, systemic autoimmune disease characterized by granulomatous inflammation and necrotizing vasculitis. While most cases occur in adults, pediatric presentations are becoming more recognized, often with atypical symptoms. We present the case of a 9-year-old girl who presented with a 3-week history of unilateral proptosis, a persistent cough, and mild fatigue. Her presentation and findings varied between infection and the inflammatory process. Histopathology of an orbital and ethmoid sinus biopsy revealed caseating and non-caseating granulomas, which further complicated the differential diagnosis. The patient also had an infection with *Streptococcus viridans, *methicillin-resistant *Staphylococcus aureus *(MRSA), and *Staphylococcus epidermidis*. Laboratory findings showed elevated inflammatory markers and a positive antineutrophil cytoplasmic antibody (ANCA) at a later stage. This case highlights the challenges in diagnosing GPA in children, particularly with an unusual presentation, and emphasizes the importance of a multi-disciplinary approach and early immunosuppressive treatment to help reduce complications from this disease.

## Introduction

Granulomatosis with polyangiitis (GPA) is a rare, small-to-medium-vessel vasculitis that commonly affects the upper and lower respiratory tracts and kidneys, and can involve multiple other organs. Although GPA predominantly affects adults, pediatric cases are increasingly being reported. Constitutional features like fever, exhaustion, weight loss, and symptoms that resemble common infectious diseases are common in children with GPA, and they frequently cause delays in diagnosis [1]. Early detection is made more difficult by the possibility that the classic signs of GPA-upper and lower airway involvement, along with glomerulonephritis, may not be evident at first. The disease progresses over weeks to months in many pediatric patients, affecting organs, especially the kidneys, which may not show any symptoms until severe damage has been done. Although orbital involvement in GPA has been extensively documented in adult populations, it is comparatively uncommon in children and is easily confused with orbital cellulitis or an odontogenic complication [2].

In this case study, a 9-year-old girl with a diagnostically difficult GPA presentation first presented with unilateral proptosis and a persistent cough, which are symptoms more frequently linked to orbital cellulitis or bacterial sinusitis. The overlapping characteristics of acute infection, granulomatous inflammation, and renal involvement that only became apparent during hospitalization masked the final diagnosis of GPA. In addition to the polymicrobial findings and unusual initial presentation, this case is noteworthy for the development of crescentic glomerulonephritis, a sign of renal GPA, and the subsequent need for immunosuppressive treatment [1].

By examining this atypical presentation and subsequent management of GPA, we aim to emphasize the importance of maintaining a broad differential diagnosis in children with persistent sinus and orbital infections unresponsive to conventional therapies. Early recognition of systemic features and prompt collaboration between specialties such as pediatrics, infectious diseases, ENT, rheumatology, and nephrology are critical for improving outcomes in pediatric GPA.

## Case presentation

A 9-year-old girl of Southeast Asian ethnicity presented with an extensive clinical history that spanned several weeks, marked by persistent fever, right eye swelling, cough, and poor oral intake. In the first week of illness, the patient was treated for a suspected viral upper respiratory infection and bronchitis with symptomatic management. However, due to concern for possible bacterial infection, empiric antibiotics were started. When her symptoms, including persistent fever, right eye proptosis, and productive cough, worsened despite the antibiotics, she was admitted for further evaluation. Laboratory results revealed elevated C-reactive protein (CRP) (161 mg/L), indicating significant systemic inflammation. Upon admission, the child’s symptoms persisted, with ongoing fever, progressive right eye swelling, and a productive cough. A dental examination suggested a potential source of infection in the right maxillary molar, prompting an extraction of the right maxillary tooth.

She was brought to our facility by her parents after she showed no improvement in symptoms. Initial assessment along with a CT scan of the sinuses, revealed pansinusitis with involvement of the right maxillary, ethmoidal, and frontal sinuses, as well as evidence of orbital cellulitis with right-sided proptosis. The extent of the infection, coupled with the patient’s failure to improve despite treatment, led the clinical team to suspect a more complex underlying condition.

A pan sinus navigation and right medial orbital wall decompression was done by the Otorhinolaryngology team, and biopsies were taken from the orbit, maxillary, and ethmoid sinuses.

**Figure 1:**
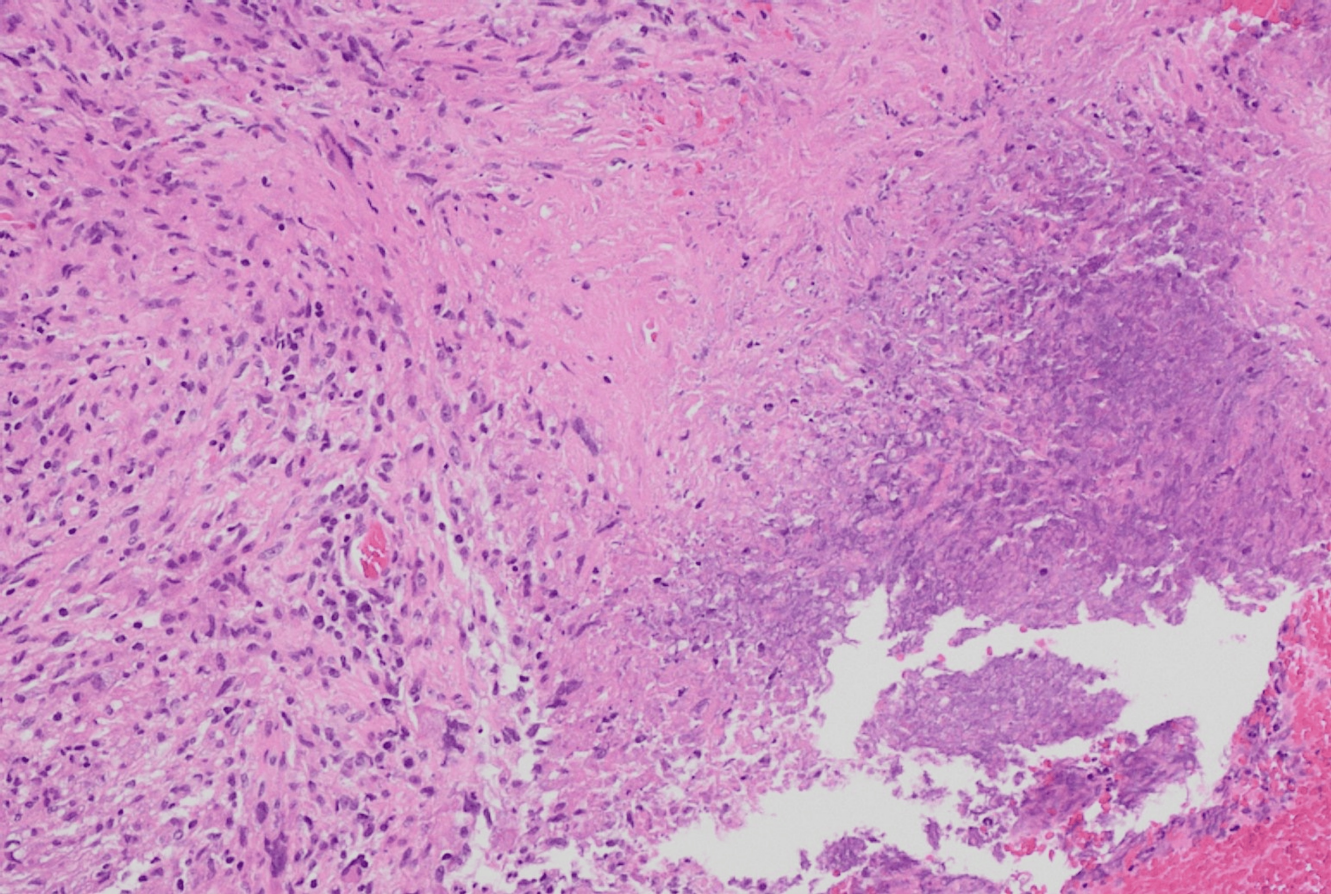
Biopsy from right orbital tissue showing caseating necrosis.

**Figure 2:**
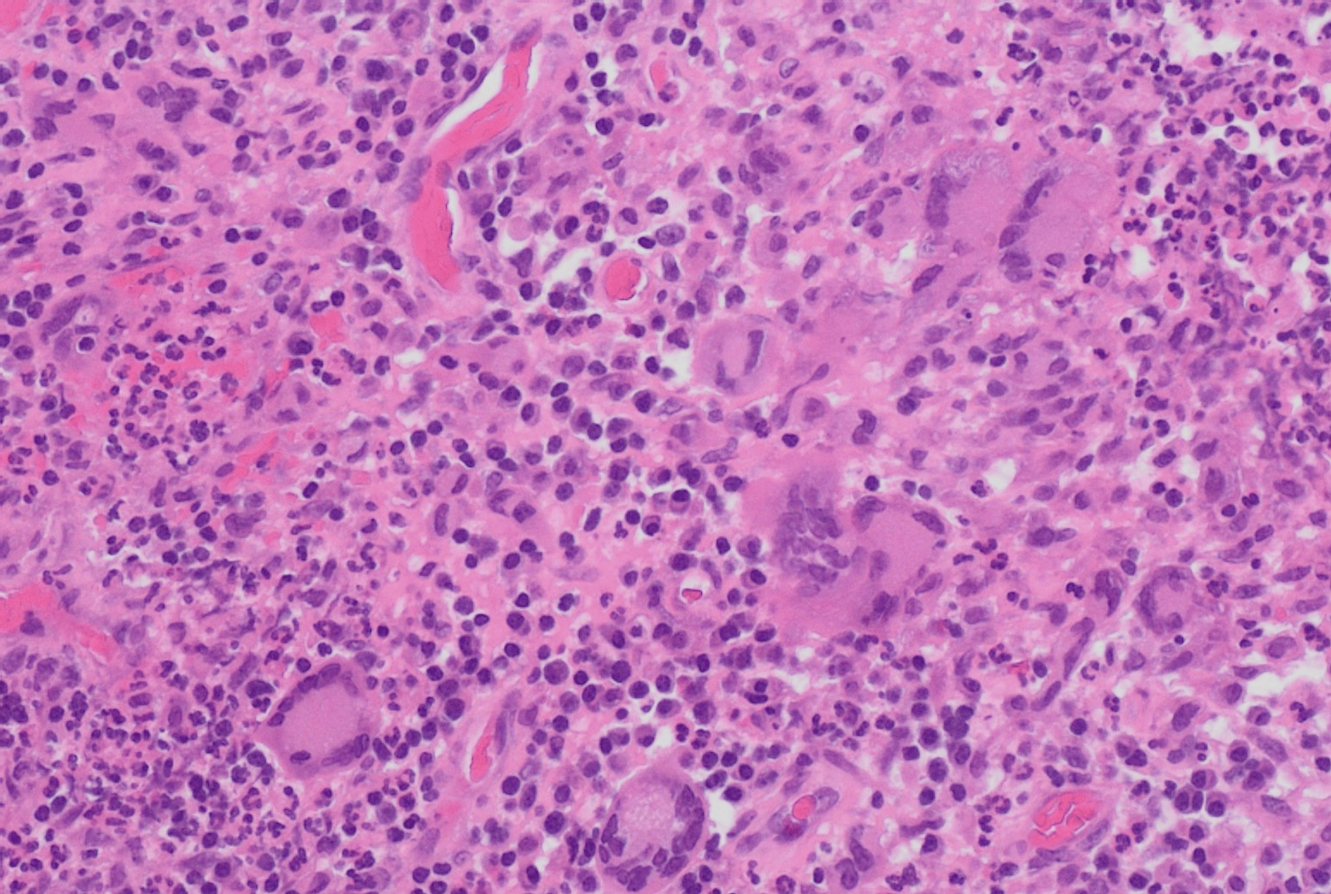
Biopsy from the maxillary sinuses showing granulomatous inflammation with the presence of giant cells.

Histopathological examination of biopsied tissues from the right maxillary, ethmoidal, and orbital regions revealed non-caseating granulomas with giant cells in the sinuses and caseating granulomas in the orbital tissue, which can be seen in Figure 1 and Figure 2, respectively. Due to the mixed characteristics of the granulomas, the differential diagnosis at this stage was broad. Possible differentials included atypical infections such as tuberculosis and fungal infections, as well as autoimmune conditions, all of which complicated the clinical picture.

Cultures taken from nasal and ocular swabs showed growth of methicillin-resistant *Staphylococcus aureus* (MRSA) in the nasal passages and *Staphylococcus epidermidis* in the ocular swab, suggesting possible infections or colonization by these organisms. *S. epidermidis*, a common skin commensal, was likely a contaminant but may have contributed to the patient’s ocular findings. However, the most concerning microbial finding was the presence of *Streptococcus viridans*. This microorganism, part of the normal oral flora, was isolated both from the right maxillary tissue and orbital samples, further confirming the direct spread of infection from the upper respiratory and oral cavity to the sinuses and orbit.

The presence of *S. viridans* in the sinuses and orbit, combined with the granulomatous inflammation observed on histopathology, suggested a more complex polymicrobial infection with both bacterial and potentially autoimmune components.

Given the microbial findings, she was started on vancomycin(15 mg/kg every 12 hours) to target MRSA, along with levofloxacin to provide coverage for other potential pathogens. She was also treated with mupirocin nasal ointment and chlorhexidine washes to decolonize MRSA from the nasal passages and skin.

A repeat CT scan of the sinuses revealed persisting pansinusitis, with persistent proptosis and orbital cellulitis in the right eye. The inflammation was extensive, particularly in the right maxillary, ethmoidal, and frontal sinuses, where mucosal enhancing soft tissue and fluid accumulation suggested acute sinusitis. This condition caused a blockage of the orbital meatal complex on the right side and extended to the medial and inferior rectus muscles, with proptosis observed, raising concern for orbital cellulitis.

**Figure 3:**
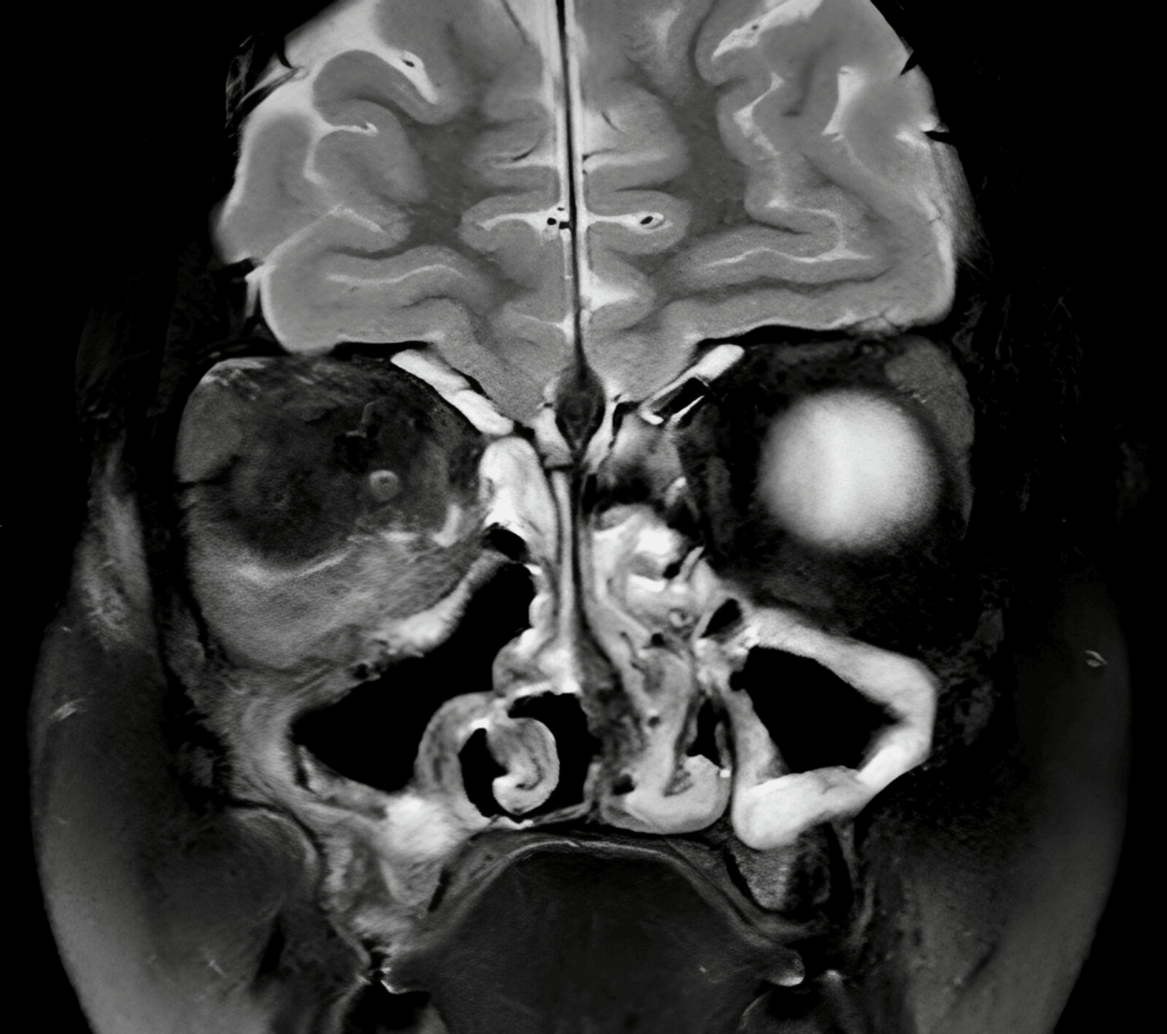
MRI Brain showing widespread inflammation around the right orbit and bilateral nasal sinuses

**Figure 4:**
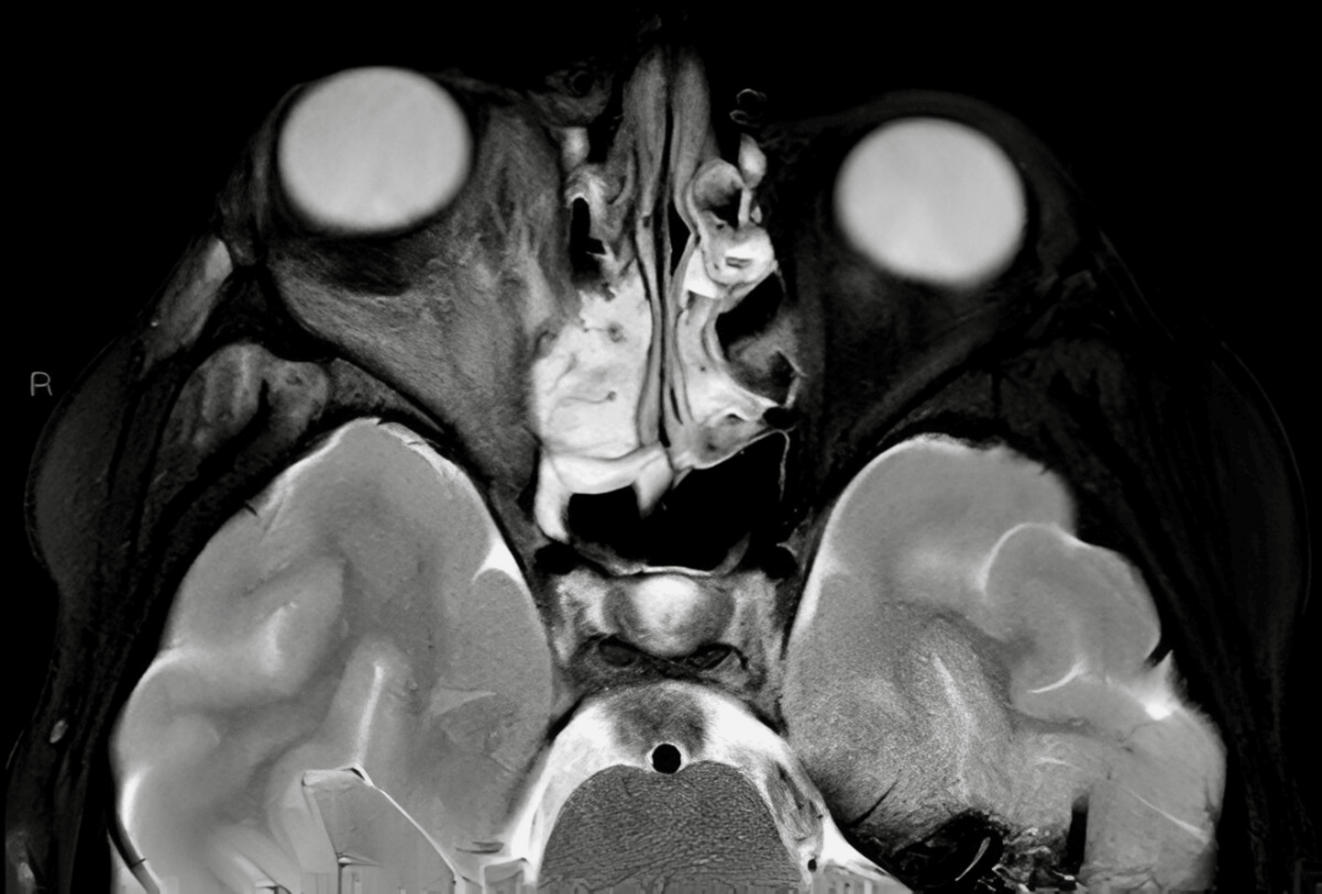
T2-weighted MRI of the brain showing right sided proptosis and widespread inflammation

**Figure 5:**
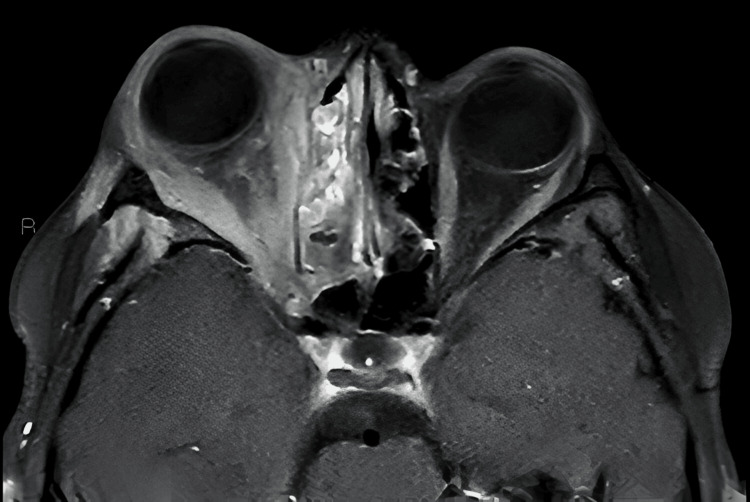
Post-contrast T1-weighted MRI of the Brain demonstrating right-sided proptosis with extensive periorbital and orbital inflammation

The MRI brain confirmed significant inflammation of the right orbit, involving both pre- and post-septal regions seen in Figures 3-5. There was no evidence of intracranial extension, but mild inflammation was noted in the right masticatory space and lateral pterygoid. Eye swab taken showed only normal flora, and CRP levels were elevated at 59, indicative of active inflammation but without evidence of a more severe bacterial infection.

**Figure 6:**
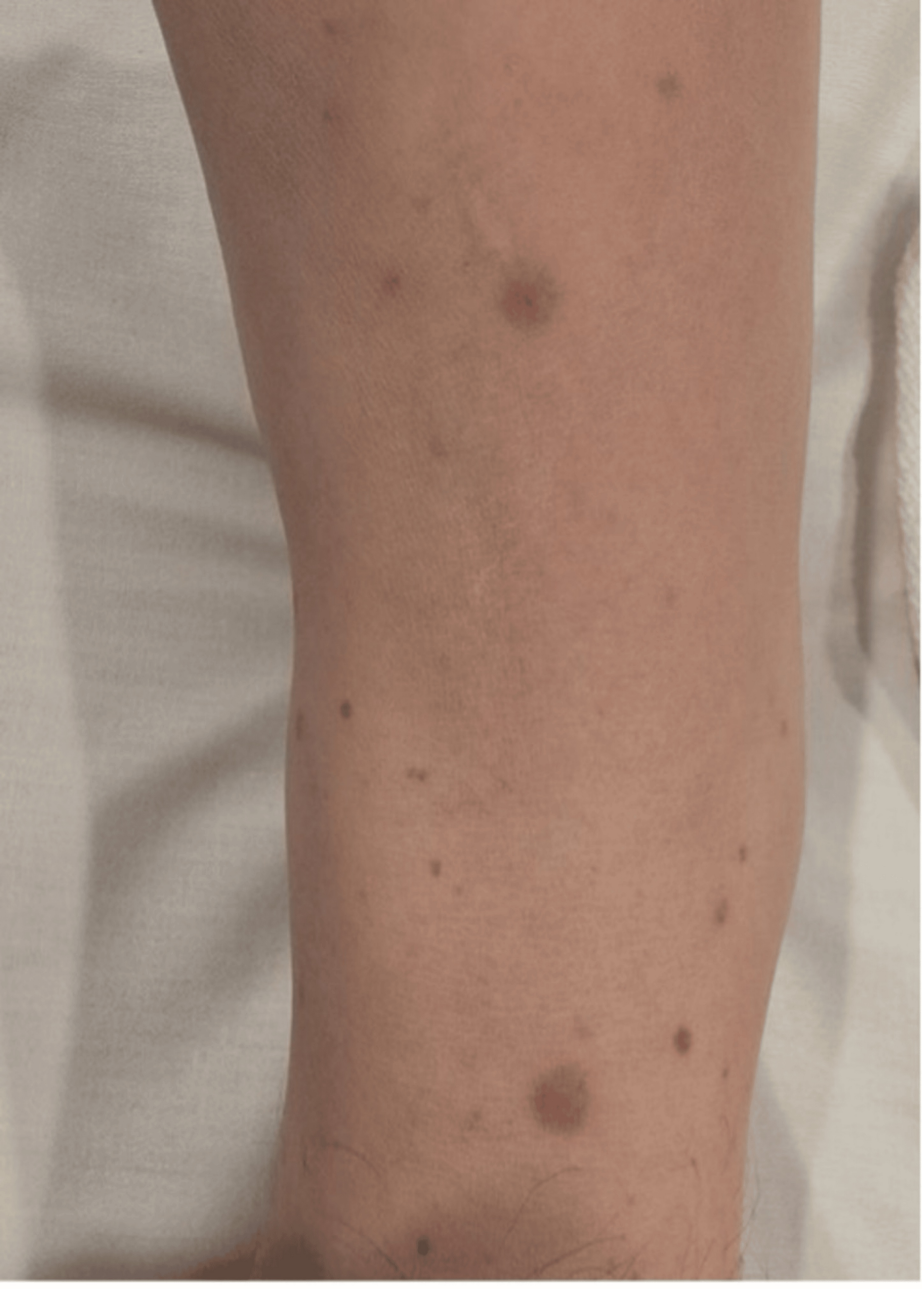
Purpuric spots on the lower part of the leg

During the pediatric pulmonology consultation, further investigations were carried out to exclude the possibility of tuberculosis and to assess the potential for an autoimmune cause. CT scans of the thorax and abdomen were normal. Laboratory tests for ANA, perinuclear staining antineutrophil cytoplasmic antibody (p-ANCA), c-ANCA, tuberculosis, and sarcoidosis were performed, and she was re-admitted to our facility. Additionally, she developed a diffuse, non-blanching rash on the dorsal side of her lower limbs, seen in Figure 6, prompting a consultation with a dermatologist, who suspected Henoch-Schönlein purpura (HSP), which added more complexity to the case.

The c-ANCA testing returned strongly positive, confirming the diagnosis of GPA. Further investigation revealed 3+ proteinuria and 4+ microscopic hematuria, suggesting significant renal involvement. The urine albumin-to-creatinine ratio was markedly elevated at 2,528.26 mg/g (normal: 0-30 mg/g), indicating substantial glomerular injury. In addition, ferritin levels were markedly elevated (1,200 ng/mL; normal range 20-200 ng/mL), and the erythrocyte sedimentation rate (ESR) was elevated at 72 mm/h.

Methylprednisolone at a high dose (15 mg/kg/day) was administered to the patient for three days in order to control the acute inflammatory reaction after virtual discussions with a pediatric rheumatologist. She was discharged with a high dose of oral corticosteroids and advised to consult a pediatric rheumatologist for further treatment.

**Figure 7:**
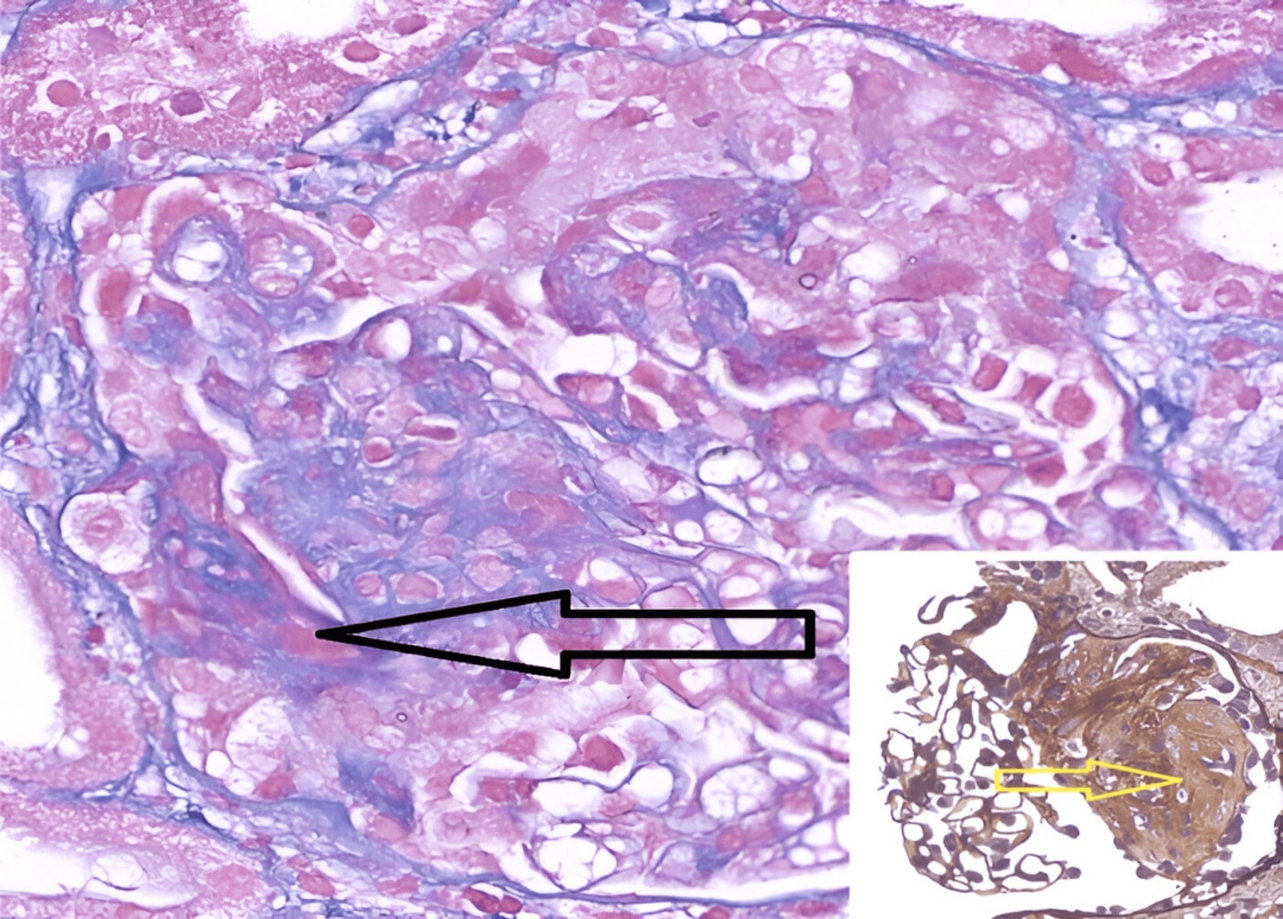
Renal Biopsy showing pauci-immune necrotizing crescentic glomerulonephritis with segmental sclerosis

Subsequently, a renal biopsy (Figure 7) confirmed pauci-immune necrotizing crescentic glomerulonephritis, with 50% active crescents and acute tubular injury - a hallmark of GPA-related renal involvement and upon the recommendations of the pediatric rheumatologist and nephrologist, she received rituximab, a monoclonal antibody targeting CD20+ B cells, which is effective in treating GPA. 

**Figure 8:**
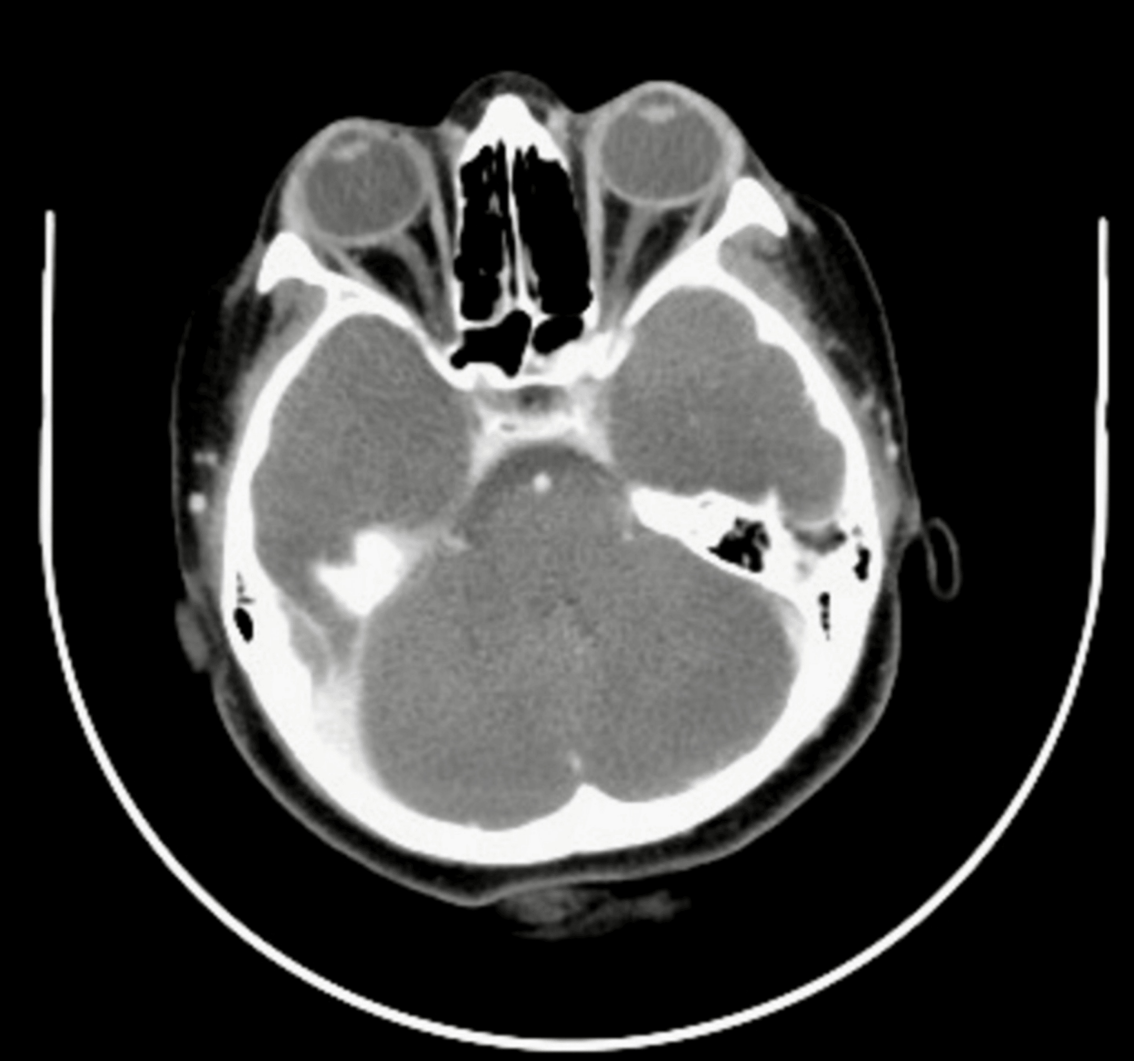
Repeat CT scan showing resolution of ocular proptosis and inflammation

After initiating the course of high-dose corticosteroids, the patient’s fever resolved, and her right eye swelling gradually improved. However, the proteinuria (4+) and microhematuria (4+) persisted on follow-up urinalysis, indicating ongoing renal involvement despite improvement in other clinical parameters. Renal function was closely monitored, and the patient was discharged with oral prednisone (50 mg daily) for maintenance, enalapril (2.5 mg twice daily) for blood pressure control and renal protection, and calcium (500 mg daily) and vitamin D supplements to manage the risk of osteoporosis from long-term steroid use. At her most recent follow-up, a repeat CT scan confirmed resolution of her ocular and nasal sinus symptoms (Figure 8); however, she continues to have 1+ proteinuria, and her c-ANCA titers, though reduced, remain positive. It is important for her titers to become negative after induction due to the increased possibility of relapse in patients with GPA. Her ongoing treatment with rituximab will help further alleviate her symptoms and reduce the risk of disease relapse. 

## Discussion

GPA in pediatric patients is a rare, complex condition that poses significant diagnostic and therapeutic challenges. The incidence rate of GPA in the pediatric population is 1.8 per 1,000,000, compared to 12 per 1,000,000 in adults. The reported prevalence of GPA across all age groups varies between 2 and 146 cases per million individuals, with a significantly lower incidence in children [3].

Although the disease is more common in adults, its presentation in children can vary and often requires a high index of suspicion due to overlapping symptoms with other conditions. This case report highlights a pediatric patient with GPA who presented with proptosis and infections caused by *S. viridans* and MRSA, adding complexity to the clinical picture. We have also reviewed the literature for GPA in pediatric patients, emphasizing its clinical presentation, diagnostic difficulties, management approaches, and the impact of infectious complications on disease progression.

The typical clinical presentation of GPA involves a triad of upper airway, lower respiratory tract, and renal manifestations. However, symptoms often develop gradually and may be nonspecific, including dyspnea, chronic cough, fever, malaise, fatigue, and weight loss [3]. Our patient initially presented with symptoms suggestive of common upper respiratory infections. However, further workup revealed multisystem involvement, including ocular proptosis and infection with *S. viridans* and MRSA. Notably, the patient showed no initial symptoms of kidney disease such as oliguria, edema, gross hematuria, or hypertension, which contributed to the delay in confirming the diagnosis.

Proptosis, a rare but significant manifestation of GPA, typically arises from granulomatous inflammation of the orbital tissues [2]. This orbital involvement can mimic isolated ocular issues or infections, complicating the diagnosis of GPA. While GPA can manifest in various ways, including episcleritis, proptosis, scleritis, uveitis, conjunctivitis, and corneal ulceration, studies suggest that scleritis is the most frequent ocular manifestation at diagnosis, followed by proptosis and uveitis [4].

The patient also experienced multiple severe infections, including *S. viridans* and MRSA. These infections added complexity to the clinical picture, as these pathogens are not usually linked with GPA. This led to an initial misdiagnosis, compounded by limited clinical history provided by the parents and additional complicating factors such as a concurrent tooth infection. *S. viridans* can often be associated with mixed infections, particularly in the setting of prolonged inflammation or disruption of mucosal barriers, as seen in this case. The *S. viridans* found in the orbital and maxillary samples raised concerns about a potential oral cavity-related source of the infection, although the main concern was the granulomatous inflammation linked to GPA.

Infections in GPA patients, especially those on immunosuppressive therapy, are a significant concern. As noted in the literature, the immunosuppressive agents used to treat GPA, including corticosteroids and rituximab, can predispose patients to opportunistic infections, making prompt recognition and treatment of infections essential [1,5].

There may also be a variety of cutaneous manifestations, the most common being purpura [6]. In certain case reports, patients exhibited palpable purpuric lesions and renal involvement, initially suspected to be manifestations of IgA vasculitis. However, further investigation revealed positive c-ANCA and findings from sinus and renal biopsies, ultimately leading to a diagnosis of GPA [5].

Similarly, in our patient, the presence of palpable purpuric lesions prompted us to expand our differential diagnosis and consult with other specialists. The results from the biopsies and c-ANCA testing were instrumental in confirming the final diagnosis.

Prior to the widespread availability and use of ANCA testing, the 1990 American College of Rheumatology (ACR) classification criteria for vasculitis were developed. Based on the updated American College of Rheumatology and European Alliance of Associations for Rheumatology (ACR/EULAR) (2022) [3] clinical classification criteria, our patient exhibited nasal involvement (+3), laboratory criteria showed a positive c-ANCA (+5) and biopsy revealed caseating and non-caseating granulomas, along with granulomatous inflammation (+2). She also had pauci-immune glomerulonephritis on biopsy (+1) and sinusitis, diagnosed clinically and on imaging (+1). These findings resulted in a total score of 12, exceeding the threshold of 5 and further supporting the diagnosis of GPA.

The treatment of pediatric GPA is centered around immunosuppressive therapy, with corticosteroids typically used as first-line therapy. However, in severe cases or those with high relapse rates, rituximab-a monoclonal antibody targeting CD20+ B cells-has become an important alternative [7]. A combination of corticosteroids, cyclophosphamide, and rituximab to suppress the immune system and induce remission. After remission is achieved, patients are monitored with a maintenance dose of steroids and other disease-modifying therapies. Early diagnosis of GPA is crucial for patient survival, as the condition has a one-year mortality rate of nearly 90% if untreated [8].

Rituximab has been found to be effective in inducing remission and preventing relapse in pediatric GPA patients, as shown in multiple case reports [8,9]. Our patient was started on high-dose corticosteroids in combination with rituximab following the diagnosis of GPA. The use of rituximab, spaced over three infusions, two weeks apart, was aimed at achieving remission and preventing disease flare-ups.

Given the patient's existing crescentic glomerulonephritis, close monitoring of renal function was essential. Due to the potential for rapid progression, a recognized complication of GPA [2], serial serum creatinine levels and urinalysis were performed to monitor renal status and assess for any evidence of decline.

The prognosis for pediatric GPA depends on the severity of organ involvement and the response to treatment. Early diagnosis and aggressive immunosuppressive therapy improve the chances of achieving remission and reducing the risk of long-term organ damage. Our patient’s prognosis is favorable, as the use of rituximab has been associated with a high rate of remission in pediatric cases, although long-term follow-up will be essential to monitor for potential relapses [9].

Infections can be exacerbated by the immunosuppressive treatments used in GPA, and patients with GPA are at increased risk of recurrent or chronic infections. Careful monitoring for signs of infection and timely intervention are key to managing these risks [10]. The long-term prognosis for pediatric patients with GPA is largely influenced by the severity of renal involvement, as early renal damage may lead to complications such as the need for dialysis or renal transplantation. Therefore, early diagnosis and timely intervention are crucial to prevent complications and improve patient outcomes [11].

## Conclusions

This case highlights the complexity of GPA in a pediatric patient, presenting with a wide differential diagnosis that included atypical bacterial infections like tuberculosis, fungal infections, as well as autoimmune conditions, which was further complicated by the coexisting dental issues. The identification of MRSA, *S. epidermidis*, and *S. viridans* in different anatomical sites was likely due to superadded infection, further complicating the clinical picture. There was also significant multisystem involvement, including sinusitis, orbital cellulitis, and nephritis, which helped piece together this puzzling case. Prompt recognition of GPA, along with high-dose steroids and rituximab, helped control the disease, although renal involvement remains a concern. The presence of granulomatous inflammation, positive c-ANCA results, and elevated urinary markers confirmed the diagnosis and guided the treatment plan. Ongoing monitoring for renal function and disease relapse is crucial, given the severity of her presentation and the potential for long-term complications.
